# Serum and Urinary Osteocalcin in Healthy 7- to 19-Year-Old Finnish Children and Adolescents

**DOI:** 10.3389/fped.2021.610227

**Published:** 2021-08-24

**Authors:** Päivi M. Paldánius, Kaisa K. Ivaska, Outi Mäkitie, Heli Viljakainen

**Affiliations:** ^1^Children's Hospital, University of Helsinki and Helsinki University Hospital, Helsinki, Finland; ^2^Research Program for Clinical and Molecular Metabolism, University of Helsinki, Helsinki, Finland; ^3^University of Turku, Institute of Biomedicine, Turku, Finland; ^4^Department of Molecular Medicine and Surgery, Center for Molecular Medicine, Karolinska Institutet, and Clinical Genetics, Karolinska University Hospital, Stockholm, Sweden; ^5^Folkhälsan Research Center, Helsinki, Finland; ^6^Department of Food and Nutrition, University of Helsinki, Helsinki, Finland

**Keywords:** pediatric normative values, bone turnover markers, serum osteocalcin, urinary mid-fragment osteocalcin, sex and age-specific ranges

## Abstract

Children and adolescents have high bone turnover marker (BTM) levels due to high growth velocity and rapid bone turnover. Pediatric normative values for BTMs reflecting bone formation and resorption are vital for timely assessment of healthy bone turnover, investigating skeletal diseases, or monitoring treatment outcomes. Optimally, clinically feasible measurement protocols for BTMs would be validated and measurable in both urine and serum. We aimed to (a) establish sex- and age-specific reference intervals for urinary and serum total and carboxylated osteocalcin (OC) in 7- to 19-year-old healthy Finnish children and adolescents (*n* = 172), (b) validate these against standardized serum and urinary BTMs, and (c) assess the impact of anthropometry, pubertal status, and body composition on the OC values. All OC values in addition to other BTMs increased with puberty and correlated with pubertal growth, which occurred and declined earlier in girls than in boys. The mean serum total and carboxylated OC and urinary OC values and percentiles for sex-specific age categories and pubertal stages were established. Correlation between serum and urinary OC was weak, especially in younger boys, but improved with increasing age. The independent determinants for OC varied, the urinary OC being the most robust while age, height, weight, and plasma parathyroid hormone (PTH) influenced serum total and carboxylated OC values. Body composition parameters had no influence on any of the OC values. In children and adolescents, circulating and urinary OC reflect more accurately growth status than bone mineral density (BMD) or body composition. Thus, validity of OC, similar to other BTMs, as a single marker of bone turnover, remains limited. Yet, serum and urinary OC similarly to other BTMs provide a valuable supplementary tool when assessing longitudinal changes in bone health with repeat measurements, in combination with other clinically relevant parameters.

## Introduction

Normal pediatric reference ranges for bone turnover markers (BTMs), reflecting bone formation and resorption, are a prerequisite for timely assessment of metabolic bone disorders and monitoring of response to therapy or disease progression (1). As compared with adults, children and adolescents have elevated circulating concentrations of BTMs, reflecting sequential periods of high growth velocity and rapid bone turnover (2, 3). The highest levels of BTMs have been described in infants and children during the first 4 years of life (2, 4, 5); thereafter, marker levels decrease slightly and remain relatively stable until peaking again at puberty (6). Puberty is a crucial period for final bone mass accrual (7), with 25% increase in lumbar spine volumetric bone mineral density (BMD) during puberty (8). Nevertheless, there appears to be no relationship between the length of puberty and accrual of peak bone mass (9).

Net gain in bone mass in childhood results from bone modeling. Even if also bone resorption takes place during this time, the bone mass accrual is driven by the rapid bone formation, which by far exceeds the rate of resorption (10). The subsequent increase in BTMs should coincide with the pubertal growth spurt while increased variation in BTM levels also demonstrates correlation with growth velocity. This fluctuation surges during pubertal years due to considerable changes in absolute measured concentrations of both formation and resorption markers with age (10–12). Generally, almost 90% of the skeletal mass is being attained by 18 years (13), even if the process greatly varies between individuals, and is influenced by many physiological and pathological factors such as genetics, gender, ethnicity, prematurity, endocrine and mechanistic factors, (mal)nutrition, and pharmacological factors (14). Consequently, any longitudinal measurement to distinguish between normal and abnormal bone turnover in a growing child necessitates comparison against pediatric normative reference curves.

BTMs are specific bone-derived proteins or their fragments, which are present in serum and/or urine, often classified as markers reflecting either bone formation or bone resorption, and their concentrations reflect bone metabolic activity in total body at a given time point. The use of a composite set of formation and resorption markers instead of single markers is recommended in the longitudinal assessment of bone metabolism, as the sensitivity and/or specificity of any single marker is usually poor (1, 15).

Osteocalcin (OC) is a bone-derived protein, a widely used marker of bone formation in adults, which has also been shown to correlate with serum testosterone and bone maturation in boys (16). Previous pediatric studies have demonstrated that serum OC is a sensitive marker of skeletal growth in healthy children and in those with pathologically increased growth velocity (17). OC is metabolized in kidneys as measurable mid-molecule fragments. The urinary levels of OC correlate well with serum OC concentrations and other serum BTMs in adults (18). Urinary mid-molecular fragments of OC have been used alone in adults as an index of bone turnover but have not yet been established as a marker of bone turnover in children and adolescents. In one study, the urinary mid-fragments predicted catch up growth in prematurely born infants suggesting that it may be a valid marker even in children (19).

Increasing adiposity has been suggested as a risk factor for fractures in children (20, 21) while the role of BMI in extremity fractures is unclear (22). Studies in young adults, with or without impaired glucose tolerance or diabetes (23–25), indicate that OC may play a potential role in energy metabolism, even if the results are inconsistent and require further validation (26). In reverse, poor glycemic and metabolic control may affect bones and/or bone cell function (27, 28). Thus, additional adjustment of BTM reference range data for metabolic factors and adiposity (BMI and body composition), as well as exclusion of individuals with diabetes or impaired glucose tolerance, provides a foundation for correct interpretation of BTM results in healthy pediatric subjects (29).

Here, we aimed to establish sex- and age-specific reference intervals for serum and urinary OC in healthy Finnish children and adolescents (29). We assessed the impact of anthropometry, pubertal status, and fat percentage and body composition on creatinine corrected urinary and serum total and serum carboxylated OC levels. Additionally, we evaluated the concentrations of standardized, validated urinary and serum BTMs reflecting bone formation and resorption for each age cohort, separately for boys and girls.

## Materials and Methods

### Participants

The original study comprised 195 children and adolescents who were included in a school-based cross-sectional study in the capital region of Helsinki (60°N), in southern Finland. As previously described (30), the participants were recruited from randomly selected school classes in one primary and one secondary school for sufficient coverage of all the age groups (7–18 years) and aiming at higher than 60% participation rate. However, no strata for inclusion of all ages and sex were being introduced. Participation in this study, which was designed to assess the relationship between Vitamin D and aspects of bone health, was voluntary and invitation letters were given by the teachers to the pupils and their parents. All those willing to participate were included.

This current study included a total of 172 subjects from the original cohort, 106 girls and 66 boys, who presented with normal BMD, i.e., whole body (WB) BMD *Z*-score between −2.0 and +2.0, and had data for clinical characteristics, including puberty stage and at least sufficient serum samples for OC analysis. One outlier (an adolescent boy) was excluded due to an unreliable test result for an exceptionally high urinary OC value.

### Ethics Statement

The Helsinki and Uusimaa Hospital District Independent Ethics Committee approved the study protocol and a written, age-adjusted informed consent was given by the participants and/or their caregivers, as appropriate. The study was carried out according to the principles of the Declaration of Helsinki.

### Clinical Characteristics

All 172 subjects were evaluated for anthropometry (weight, height, and BMI). As described elsewhere (30), the subjects completed a questionnaire on medical and fracture history, medications, overall health, age at menarche for girls, use of vitamin D, and calcium supplements, and data regarding physical activity and dietary intakes were collected with a semi-quantitative food frequency questionnaire. Height (cm) and weight (kg) were measured and compared with Finnish growth charts and height standard deviation (SD) score (height *Z*-score) was defined as deviation of height, in SD units, from the mean height for age and sex (31, 32). We included no separate analysis on ethnicity due to limited ethnic heterogeneity (>90% were ethnic Caucasians/Finns).

Based on questionnaire data, serum gonadotropin and sex steroid concentrations (estradiol and testosterone), and information on menarche, pubertal development was scored either as pre-, mid-, or post-pubertal by an experienced pediatric endocrinologist (OM) (30). A united scale was introduced by converting the available Tanner stages (33) into pre-, mid-, or post-pubertal categories as follows: Tanner stages I and II were considered as pre-pubertal, stages III and IV were considered as mid-pubertal, and stage V was considered as post-pubertal.

### Biochemistry

Blood samples and second void urine were collected between 8:00 a.m. and 10:00 a.m. after an overnight fast between November and March (winter months). Serum 25-hydroxyvitamin D (25-OHD) was assayed with high-performance liquid chromatography (HPLC, evaluated by Vitamin D External Quality Assessment Scheme, DEQAS), and plasma fasting parathyroid hormone (PTH) was assayed by an immunoluminometric method.

### Serum and Urinary OC

Serum total OC and serum γ-carboxylated OC levels were determined as previously described by two-site immunoassay (34). This method for serum total OC is based on monoclonal antibodies detecting the N-terminal mid-segment of the OC molecule. Assay for carboxylated OC detects the same fragments but prefers γ-carboxyglutamic acid (Gla) containing forms of OC, with <10% cross-reactivity to fully uncarboxylated OC (35). Synthetic peptide of human OC amino acids 1–49 (Advanced Chemtech, Louisville, KY, USA) was used as a calibrator. The reported within-assay and between-assay variations (CV) for the assays are <5 and <8% (35).

Urinary mid-fragment OC was determined with the previously described two-site immunoassay for the OC mid-fragment that uses synthetic peptide of human OC amino acids 1–43 (Advanced Chemtech, as above) as a calibrator (18). The detection limit for the urinary OC assay is 0.2 mg/L and the intra-assay and inter-assay CVs in this cohort were 1.5 and 3.4%, respectively. All the urinary OC values were corrected for urinary creatinine (nM; as analyzed according to standard protocols of the Central Laboratory of Helsinki University Central Hospital). All OC samples were measured as duplicates and simultaneously at the end of the study.

### Other BTMs

Serum intact N-terminal Propeptides of Type I Collagen (S-P1NP), C-Terminal Telopeptide of Type I Collagen (S-ICTP), and alkaline phosphatase (S-ALP) were determined per validated standard protocols of the Central Laboratory of Helsinki University Central Hospital as described elsewhere (30, 36). Concentration of N-Terminal Telopeptide of Type I Collagen (U-NTx) was measured from second void urine samples with Osteomark® NTx Urine Enzyme-linked Immunosorbent Assay (Alere Scarborough, Inc., Scarborough, USA) with intra- and inter-assay CV% < 8. The given values (nM Bone Collagen Equivalents) were corrected with urinary creatinine concentration (nM).

### Analysis of Skeletal Characteristics and Body Composition

BMD, bone mineral content (BMC), and bone area (BA) were measured with dual-energy X-ray absorptiometry (DXA, Hologic Discovery A, pediatric software, version 12.4) from the lumbar spine (LS) (L1–L4), total hip, and whole body (WB). DXA measurements were performed within 3 months of the biochemical sampling. All BMD values were transformed into *Z*-scores using the equipment-specific age- and sex-adjusted reference data for US Caucasian children (37). Body composition was assessed simultaneously with DXA to obtain lean body mass and whole-body fat mass (WB fat percentage; WB fat%). Calibration of the measurements was performed by using a spine phantom; inter-CV% for the phantom BMC, area, and BMD were 0.35, 0.21, and 0.41%, respectively. The reproducibility of the DXA measurement for bone, fat, and lean mass is 1.2, 1.9, and 0.7%, respectively, in children between 10 and 18 years of age (38).

### Statistical Analysis

Baseline demographics and all the BTMs, separately for both girls and boys, were reported as descriptive statistics with means and 95% confidence intervals (CIs), standard error of the mean, and range (minimum, maximum). Additionally, Tukey's Hinges percentiles were calculated for serum and urinary OC parameters. OC data were non-normally distributed (Shapiro–Wilk test < 0.95) requiring logarithmic transformation; back-transformed data curves and values are presented here for practical use. Tertiles of Vitamin D concentrations were used to evaluate the relationship between the 25-OHD concentrations and PTH or BTMs in a univariate analysis (ANOVA), followed by Bonferroni (normally distributed data) or non-parametric Kruskal–Wallis (not normally distributed data). Multivariable regression analysis was used to identify independent determinants influencing BTM values in the overall pooled population. The covariates included anthropometric factors and their derivates such as age, sex, weight, height, BMI, height SD, weight percentage, puberty status, PTH, serum 25-OHD, whole body fat percentage, whole body weight *Z*-score, lumbar *Z*-score, and femoral *Z*-score. Simple regression analysis was first performed to screen potential predictors for OCs and a multivariate stepwise linear regression model was used to identify and determine significant predictors for BTMs. Using the approach of stepwise variable selection, stepping up, only variables with a significance level of 0.05 were included in the model. We used SPSS for Windows 21.0 (SPSS Inc., Chicago, IL) for statistical analyses. In general, *p* < 0.05 were considered statistically significant.

## Results

### Baseline Characteristics

**Figure 1:**
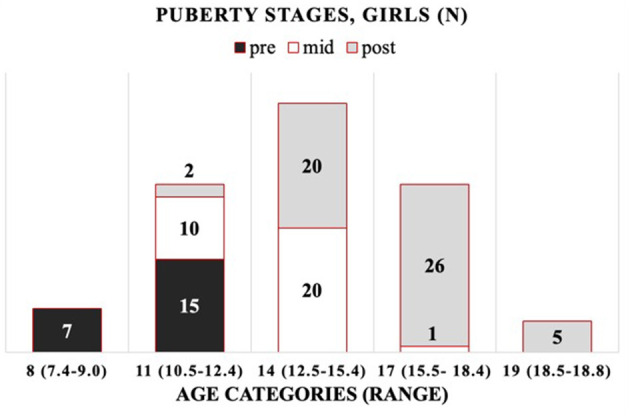
Progression of puberty status per age category for girls (*n* per puberty category).

**Figure 2:**
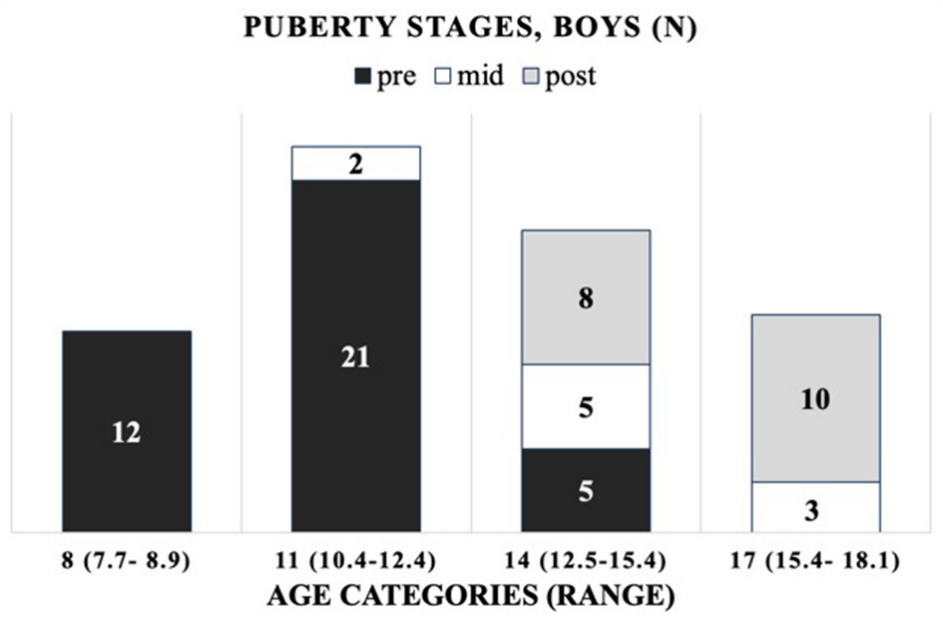
Progression of puberty status per age category for boys (*n* per puberty category).

The cohort is composed of 172 healthy Finnish children and adolescents, 106 girls and 66 boys, between 7 and 19 years of age. The mean (±SD) age was 13.8 ± 2.7 years (range 7.4–18.8) for girls and 12.5 ± 2.7 years (range 7.7–18.1) for boys (Supplementary Tables 1, 2). As the age distribution in boys differed from that in girls, including more younger subjects, we introduced robust age categories of 8, 11, 14, 17, and 19 years for girls and corresponding categories for boys, but without the oldest age category (19) (Supplementary Figure 1). For girls (G) and boys (B), the chronological age ranges per age category (minimum–maximum) are as follows: 8 years (G: 7.4–9.0; B: 7.7–8.9), 11 years (G: 10.5–12.4; B: 10.4–12.4), 14 years (G: 12.5–15.4; B: 12.5–15.4), 17 years (G: 15.5–18.4; B: 15.4–18.1), and 19 years (G: 18.5–18.8; B: none). The distribution of participants by their (pre-, mid-, and post-) pubertal stages per age category is presented in Figure 1 (girls) and Figure 2 (boys).

Baseline characteristics, including demographics and anthropometric, body composition/DXA and key biochemical parameters confirmed that the cohort represents healthy children and adolescents of normal height (Supplementary Figure 1). The background descriptive data are summarized for each age category in Supplementary Tables 1, 2 for girls and boys, respectively.

### Other BTMs

Concentrations for all the introduced BTMs, i.e., formation markers S-P1NP and S-ALP, and resorption markers S-ICTP and U-NTx, increased per age category with puberty or were already elevated prior to it. As expected, the BTM values correlated also with pubertal growth, with declining concentrations per age category observed earlier in girls (after 11 years) than in boys (after 14 years) (Tables 1A,B). The concentrations for these BTMs, for all the age categories, were within age-specific reference intervals and confirmed that the participants had a normal bone turnover at the time of evaluation.

**Table 1A:** Serum P1NP, ICTP, ALP, and urinary NTx concentrations per age category for girls.

**BTM (unit)**	**Age category (range)**	***N***	**Mean**	**SD**	**SEM**	**95% CI**		**Min**	**Max**
						**Lower bound**	**Upper bound**		
Serum P1NP (μg/L)	8 (7.4–9.0)	7	696.3	200.26	75.70	511.1	881.5	417.0	1,004.0
	11 (10.5–12.4)	27	683.7	245.68	47.28	586.5	780.9	201.0	1,095.0
	14 (12.5–15.4)	39	447.7	303.23	48.55	349.4	546.0	95.0	1,511.0
	17 (15.5– 18.4)	25	94.2	37.42	7.48	78.8	109.6	26.0	162.0
	19 (18.5–18.8)	5	68.6	23.67	10.59	39.2	98.0	43.0	95.0
Serum ICTP (μg/L)	8 (7.4–9.0)	7	15.3	3.82	1.44	11.8	18.8	11.0	20.0
	11 (10.5–12.4)	27	17.3	4.76	0.92	15.4	19.1	10.0	33.0
	14 (12.5–15.4)	39	14.9	4.58	0.73	13.4	16.4	5.0	25.0
	17 (15.5–18.4)	25	7.1	1.96	0.39	6.3	7.9	4.0	12.0
	19 (18.5–18.8)	5	5.6	0.89	0.40	4.5	6.7	5.0	7.0
ALP (μg/L)	8 (7.4–9.0)	7	248.7	77.94	29.46	176.6	320.8	122.0	343.0
	11 (10.5–12.4)	27	255.7	72.85	14.02	226.8	284.5	146.0	438.0
	14 (12.5–15.4)	38	173.1	67.50	10.95	150.9	195.3	77.0	354.0
	17 (15.5–18.4)	26	76.3	20.22	3.97	68.1	84.5	45.0	110.0
	19 (18.5–18.8)	5	58.8	14.08	6.30	41.3	76.3	39.0	74.0
Urinary NTx (nmol/mol)	8 (7.4–9.0)	7	800.9	237.73	89.86	581.0	1,020.7	617.0	1,290.4
	11 (10.5–12.4)	25	840.2	309.60	61.92	712.4	968.0	448.0	1,509.2
	14 (12.5–15.4)	33	449.4	247.82	43.14	361.5	537.2	75.4	973.8
	17 (15.5–18.4)	24	117.8	65.50	13.37	90.1	145.4	32.4	305.3
	19 (18.5–18.8)	4	89.3	14.75	7.38	65.9	112.8	68.7	101.5

**Table 1B:** Serum P1NP, ICTP, ALP, and urinary NTx concentrations per age category for boys.

**BTM (unit)**	**Age category (range)**	***N***	**Mean**	**SD**	**SEM**	**95% CI**		**Min**	**Max**
						**Lower bound**	**Upper bound**		
Serum P1NP (μg/L)	8 (7.7–8.9)	12	550.6	129.56	37.40	468.26	632.90	338.0	849.00
	11 (10.4–12.4)	23	590.3	189.84	39.59	508.17	672.36	392.0	1,266.0
	14 (12.5–15.4)	17	675.4	226.45	54.92	558.92	791.78	409.0	1,169.0
	17 (15.4–18.1)	13	276.8	179.02	49.65	168.66	385.03	97.0	581.00
Serum ICTP (μg/L)	8 (7.7–8.9)	12	14.9	2.94	0.85	13.05	16.78	11.0	21.00
	11 (10.4–12.4)	23	14.5	3.58	0.75	12.97	16.07	9.0	26.00
	14 (12.5–15.4)	17	18.2	4.35	1.05	15.94	20.41	12.0	24.00
	17 (15.4–18.1)	13	13.6	6.68	1.85	9.58	17.65	5.0	29.00
ALP (μg/L)	8 (7.7–8.9)	12	217.8	62.12	17.93	178.28	257.22	135.0	334.00
	11 (10.4–12.4)	23	241.0	55.35	11.54	217.02	264.89	168.0	391.00
	14 (12.5–15.4)	18	254.8	56.06	13.21	226.83	282.71	178.0	370.00
	17 (15.4–18.1)	13	136.2	69.80	19.36	94.05	178.41	58.0	290.00
Urinary NTx (nmol/mol)	8 (7.7–8.9)	12	670.8	279.91	80.80	492.96	848.66	291.3	1,062.0
	11 (10.4–12.4)	22	649.1	178.50	38.06	569.98	728.27	351.6	1,167.3
	14 (12.5–15.4)	15	632.0	327.63	84.59	450.52	813.38	336.7	1,302.0
	17 (15.4–18.1)	13	220.3	156.40	43.38	125.77	314.80	55.0	603.57

### Serum Total and Carboxylated OC

**Figure 3:**
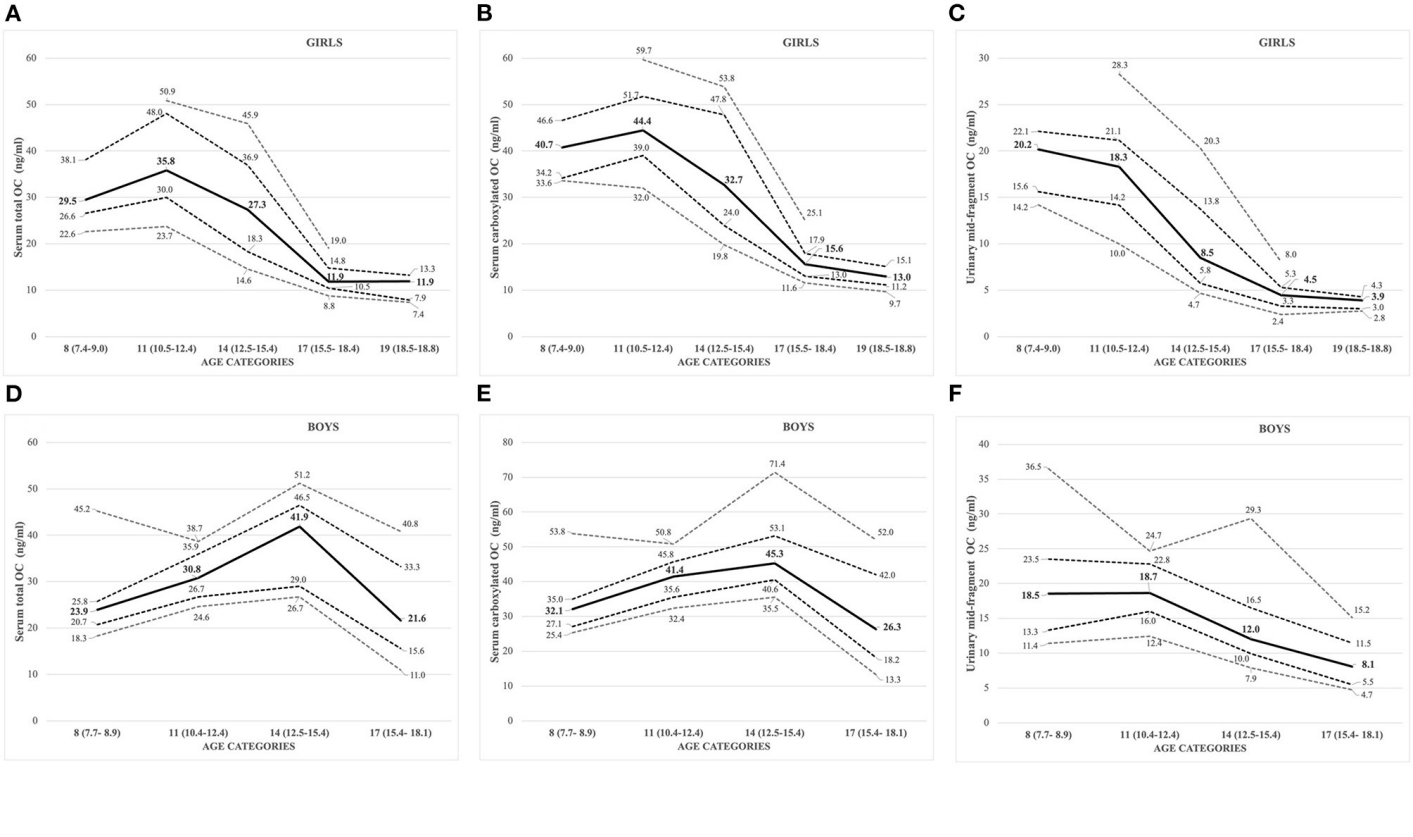
**(A–F)** Sex- and age-specific OC concentrations displayed as percentiles (10th, 25th, 50th: solid line; 75th, 90th) for serum total OC [**(A)** girls; **(D)** boys], carboxylated OC [**(B)** girls; **(E)** boys], and urinary mid-fragment OC [**(C)** girls; **(F)** boys].

All the OC concentrations associated with age and puberty (Figures 3A,B,D,E). The mean peak serum total OC values per age category were 38.7 ± 11.47 ng/ml (11 years) and 38.8 ± 9.43 ng/ml (14 years) in girls and boys, respectively. The peak OC values coincided with the mid-pubertal stage for both sexes (Figures 1, 2), and the dynamics of the opposite trends for total serum OC concentrations were most pronounced in age categories 11 to 14 in girls vs. boys. When entering the post-pubertal stage, the OC values decreased closer to those observed in young adults, and earlier in girls to levels lower vs. those observed in boys at 17 years. The pattern of concentrations of carboxylated OC was consistent with that of total serum OC (Tables 2A,B, Figures 3A–F).

**Table 2A:** Serum total, carboxylated, and urinary mid-fragment OC values as geometrical mean (±SD) and percentiles for girls.

					**Tukey's hinges percentiles**			
**Osteocalcin ng/ml**	**Age category (range)**	**Geometrical mean**	**SD**	**Minimum**	**25th**	**50th**	**75th**	**Maximum**
Serum total	8 (7.4–9.0)	33.0	10.08	22.7	25.4	29.8	35.8	52.1
	11 (10.5–12.4)	38.7	11.47	23.4	30.0	35.8	47.1	72.3
	14 (12.5–15.4)	28.6	12.03	11.3	18.4	27.3	36.9	54.9
	17 (15.5–18.4)	13.0	3.59	8.6	10.5	11.9	14.7	21.8
	19 (18.5–18.8)	10.8	2.82	7.4	8.3	11.9	12.7	13.9
Serum carboxylated	8 (7.4–9.0)	41.5	8.05	33.6	34.7	40.0	46.5	54.9
	11 (10.5–12.4)	45.8	9.45	29.3	39.9	44.4	51.6	67.6
	14 (12.5–15.4)	35.8	13.31	14.9	24.4	32.7	47.6	63.5
	17 (15.5–18.4)	16.6	4.80	10.4	13.1	15.6	17.7	28.8
	19 (18.5–18.8)	13.1	2.36	9.7	12.7	13.0	14.1	16.2
Urinary mid-fragment	8 (7.4–9.0)	21.1	5.43	14.2	18.1	21.6	22.1	31.5
	11 (10.5–12.4)	18.3	6.12	8.8	14.2	18.3	21.1	33.2
	14 (12.5–15.4)	10.5	5.89	2.4	5.8	8.5	13.7	25.6
	17 (15.5–18.4)	4.6	1.83	1.8	3.4	4.5	5.2	8.8
	19 (18.5–18.8)	3.7	0.69	2.8	3.2	3.9	4.2	4.3

**Table 2B:** Serum total, carboxylated, and urinary mid-fragment OC values as geometrical mean (±SD) and percentiles for boys.

					**Tukey's hinges percentiles**			
**Osteocalcin (ng/ml)**	**Age category (range)**	**Geometrical mean**	**SD**	**Minimum**	**25th**	**50th**	**75th**	**Maximum**
Serum total	8 (7.7–8.9)	25.5	8.88	18.0	20.9	23.9	25.6	51.6
	11 (10.4–12.4)	31.5	6.18	17.8	27.3	30.8	35.6	46.4
	14 (12.5–15.4)	38.8	9.43	26.7	29.0	39.6	46.5	53.0
	17 (15.4–18.1)	23.6	10.43	10.3	15.7	21.6	31.7	43.8
Serum carboxylated	8 (7.7–8.9)	33.6	9.39	25.4	27.2	32.1	34.9	60.1
	11 (10.4–12.4)	40.6	6.84	26.1	35.7	41.4	44.9	52.9
	14 (12.5–15.4)	48.2	11.81	31.6	40.6	45.3	52.0	71.9
	17 (15.4–18.1)	29.4	14.03	12.2	18.8	26.3	38.3	52.8
Urinary mid-fragment	8 (7.7–8.9)	19.8	8.19	11.1	13.6	18.5	22.8	39.7
	11 (10.4–12.4)	19.1	4.89	8.8	16.3	18.7	22.6	29.8
	14 (12.5–15.4)	15.2	7.66	6.9	10.6	12.1	16.1	32.3
	17 (15.4–18.1)	8.7	3.65	4.7	5.5	8.1	9.5	16.0

Based on these values, we established pediatric normative reference values for serum total and carboxylated OC per age categories, for girls and boys in Tables 2A,B, respectively. Additionally, Figures 3A,B for girls and Figures 3D,E for boys display the different percentiles (between 10th and 90th) per age category for serum total and carboxylated OC concentrations.

### Urinary Mid-Fragment OC

Contrary to the other BTMs (standard serum and urinary BTMs, and serum OC), the highest concentrations for creatinine corrected urinary mid-fragment OC values were observed in pre-pubertal children and in the youngest age category (8 years) in both girls and boys (Figures 3C,F, respectively). In general, there was a strong correlation between the serum total and/or carboxylated OC and urinary mid-fragment OC concentrations in the overall pooled cohort for girls (Spearman correlation *r* between 0.76 and 0.78, all *p* <0.001). However, for boys, the pooled urinary mid-fragment concentrations lacked correlation between total serum OC and carboxylated OC concentrations (*r* = 0.12 and 0.234, for total and carboxylated OC, respectively, *p* > 0.05). In boys, the correlation between the total serum and carboxylated and urinary OC improved progressively with increasing age, and the strongest correlations were observed in the oldest age (17) category (correlation with urinary OC: *r* = 0.692, *p* = 0.009 for total OC and *r* = 0.610, *p* = 0.027 for carboxylated OC).

### Impact of Vitamin D Status and PTH on BTM Concentrations

Serum samples for assessment of Vitamin D status were available for almost all participants: The mean serum 25-OHD (±SD) concentration was 42.5 ± 12.5 nmol/L (range 17.0–82.0 nmol/L) for all girls and boys (pooled, *n* = 171) and below 50 nmol/L in 70% (120 out of 170 subjects) of the cohort, indicating high prevalence of Vitamin D insufficiency. An inverse association between 25-OHD and the levels of both serum total and carboxylated OC was only observed in the highest 25-OHD tertile (*r* = −0.321, *p* = 0.016 for total OC and *r* = −0.381, *p* = 0.004, for carboxylated OC). Independent of the 25-OHD tertile, other assessed BTMs were strongly associated with total, carboxylated, and urinary OC (*p* < 0.001 for most BTMs, only ICTP not being associated with urinary mid-fragment OC in the highest 25-OHD tertile).

There was a negative correlation between PTH concentrations and 25-OHD tertiles (*p* = 0.014). The serum total and carboxylated OC concentrations were associated with PTH in the lowest 25-OHD tertile (Spearman, *r* = 0.404, *p* = 0.003, for total OC; and *r* = 0.384, *p* = 0.005, for carboxylated OC) but not in the entire cohort.

### Individual Determinants of Serum and Urinary OC Concentrations

We tested factors influencing the serum total, carboxylated, and urinary mid-fragment OC with multivariable linear regression model. Independent determinants for each OC parameter varied; the urinary OC was the most robust and only influenced by age and puberty, while age, height and weight (marginally for total OC), and PTH (for carboxylated OC) were significant determinants of serum OC values (Supplementary Table 3). Body fat%, BMI, or other evaluated anthropometric, DXA, or biochemical markers had no statistically significant influence on serum or urinary OC values (for all, *p* > 0.05).

## Discussion

We report, for the first time, a composite set of pediatric reference values for healthy Caucasian children and adolescents for serum total and carboxylated OC and urinary mid-fragment OC. In these young individuals with normal bone health, we demonstrated that age, height, weight, puberty, and PTH are all significant and independent determinants of serum and urinary OC levels during childhood and adolescence. Key markers reflecting overall health or growth status, such as weight and height-adjusted weight, BMI, height SD, or 25-OHD levels, seem to have no or limited significance to OC. Similarly, and rather unexpectedly, the body composition in terms of fat% or BMD *Z*-score in WB, femoral, or LS showed limited or no association with OC values. While, in adults, BTMs predict BMD values, in children, none of the measured BTMs associated with the BMD *Z*-score.

### External Validation

Our post-pubertal OC results are comparable or in line with the previously reported mean OC levels in two different cohorts of Finnish young adults (23, 25), providing an additional external validation of these data in other reference populations close to their peak bone mass. We also demonstrated a well-established pattern of variation and correlation of the BTM levels with pubertal growth; progressive increase in early puberty and the values for girls declining a few years earlier than for the boys, even if there were differences between some of the evaluated BTMs.

The pattern of dynamics differs for each BTM and may depend on physiological factors that affect these. The turnover marker concentrations also differ regarding which dimension of the bone turnover cycle they reflect. Urinary OC fragments may be derived from new biosynthetic OC or released during degradation of old matrix during bone resorption while circulating OC is being released from mature osteoblasts during late stages of bone formation (39). As bone modeling is predominantly driving skeletal growth in children and adolescents, the shift to a more pronounced pattern of coupled remodeling in post-pubertal individuals may explain the observed improved correlation between serum and urinary OC in our analysis at time points closer to the age of peak bone mass and sexual maturation. In addition, the OC levels in general were strongly driven by age. As age and pubertal stage are closely linked, this progressive increase in correlation between serum and urinary OC values can also be observed when the results are displayed by pubertal stages, reflecting the activation of hormonal maturation in both sexes rather than by age category *per se*.

### Impact of Vitamin D (25-OHD) Status on Markers of Bone Turnover

Bone mineralization as a process is dependent on adequate dietary intake of calcium and phosphate. In turn, the absorption of calcium is dependent on the intake and levels of active Vitamin D (40). However, controlled studies in adolescents show no relationship between serum 25-OHD and calcium absorption efficiency with higher 25-OHD concentrations (41), but in those with serum 25-OHD concentrations below 50–62.5 nmol/L, as in our cross-sectional cohort, the relationship appears negative (30). In a Vitamin D sufficient state, net calcium absorption is ~30%, and during active growth in pediatric populations, it increases to 60–80% (42). Vitamin D deficiency leads to diminished levels of calcium and phosphate, the essential constituents for bone mineralization (43). The relationship between Vitamin D and calcium absorption efficiency has not been studied extensively in children and adolescents and only few studies have explored the effect of the nutritional status, especially of 25-OHD on the levels of BTMs, in the otherwise healthy children.

At the time of study, 25-OHD levels were even lower than expected in our healthy pediatric population (mean value, 42.5 nmol/L), despite the reported use of supplements in addition to dietary intakes that were in accordance with the national recommendations (30). More recently, the Vitamin D status in the adult population has significantly improved due to fortification of commonly used foods with Vitamin D and increase in Vitamin D supplementation (44). Nevertheless, investigation of association between 25-OHD status, but also potentially even BMD *Z*-score, BMC, bone area, and BTMs in a healthy population, is essential for interpretation of the effect of potential sub-optimal bone mineralization on any BTM results.

### Adjusting Local Reference BTM Values

Reliable assessment of pediatric reference data for BTMs requires a representative sample population of healthy children and adolescents within a well-defined geographical area. Many factors show geographical variation and influence BTM reference values, e.g., Vitamin D (in)sufficiency, dietary habits such as milk intake, local recommendations for supplementation of minerals or vitamins, exercise habits, and even religious factors such as veiling practices (45). Hence, every geographical area should ideally have access to locally adjusted reference values. Most of the available BTM data in children and adolescents are only available for *post-hoc* analysis of birth cohorts, different bone-derived diseases, osteoporosis, or conditions inducing a secondary effect of glucocorticoid therapy on their bone health (46–49). In these populations, assessment of calcium, PTH, and phosphate levels in relation to the BTMs has been demonstrated as crucial for correct interpretation of impaired bone markers and metabolism in children and adolescents. In our cohort, only PTH and 25-OHD besides age, height, and weight were shown to be independent determinants of serum total and carboxylated OC. Our results are therefore aligned with a recent cohort study, which showed that total OC together with other BTMs and hormonal regulation were mainly affected by BMI (50): emphasizing the importance of yet considering the impact of individual body weight on interpretation of biomarkers and exploring long-term consequences of obesity on bone health in increasingly overweight cohorts of children and adolescents in general.

### Impact of Sampling Methodology on Results

When assessing any biological markers in children and adolescence, a preferred sampling method is non-invasive, such as urinary sampling as an alternative to serum sampling. Yet, in our pediatric cohort, the urinary samples were less often retrievable (92 and 94% for girls and boys, respectively) than serum samples (100% for all). The mid-fragment urinary OC reflects different aspects of bone turnover, also potentially resorption (39, 51), and it has also been demonstrated to be a marker of growth in a special population of prematurely born infants during their postnatal growth spurt (19). However, our study results indicated that there were more uncertainties for use of both serum OC and urinary OC, especially in younger boys, due to serum OC in youth being mainly derived from *de novo* biosynthesis and bone formation. The improved correlation with increasing age might therefore simply be an indicator of proportionally increased presence of balanced remodeling and synchronized coupling signals.

### Strengths and Limitations of Our Analysis

The main weakness of our study was also the limited overall number of subjects as a total of 172 children and adolescents are less than recommended by international guidelines for validated assessment of reference intervals (4). In particular, the low number of boys with advanced pubertal stage might partially explain the low correlation between the serum and urinary OC values in growing boys. On the other hand, assessment of both serum and urinary OC values provided valuable information related to the different roles of these two OC components that possibly reflect both bone formation and resorption (52). We also targeted anthropomorphic factors, components of body composition as potential predictors of BTMs and bone health, while we did not include some of the more advanced biomarkers, such as insulin-like growth factor-1 or its binding protein, shown to predict and explain a large proportion of variation in calcium retention in adolescent boys (53). Nevertheless, we have established reference ranges for younger individuals in a relatively large cohort of healthy girls and boys, and a proposal for a range of normal values for those approaching post-pubertal ages, by presenting OC values scattered over different stages of puberty and growth spurt, with a confirmatory array of standardized BTMs reflecting bone turnover from childhood toward adulthood and peak bone mass. Our cohort displays characteristics typical for a Caucasian pediatric population located on the northern hemisphere, including low 25-OHD levels in midwinter samples, and therefore providing a platform and reference intervals for clinical interpretation of BTM results in corresponding individuals.

## Conclusions

The proposed sex- and age-specific reference intervals for serum and urine OC in healthy Finnish children and younger adolescents were mostly influenced by anthropomorphic factors such as age, puberty status, height, and weight. In younger children (age < 11), circulating OC reflects more growth status than bone metabolism *per se*. Thus, its validity, similar to other BTMs, as a single determinant of healthy pediatric bone status alone might be limited. Yet, BTMs provide a supplementary tool when assessing longitudinal changes in bone health with repeat measurements, in combination with other clinically relevant parameters.

## Data Availability Statement

The original contributions presented in the study are included in the article/Supplementary Material, further inquiries can be directed to the corresponding author/s.

## Ethics Statement

The Helsinki and Uusimaa Hospital District Independent Ethics Committee approved the study protocol and a written, age-adjusted informed consent was given by the participants and/or their caregivers, as appropriate. The study was carried out according to the principles of Declaration of Helsinki.

## Author Contributions

OM contributed to the initial acquisition of the clinical data and assessed the participants at baseline. The analytical phase of the OC assays was designed and partially conducted by PP and KI. PP and HV analyzed the results. PP drafted the first version of the manuscript. All authors contributed to the conceptual design of the current study, initial interpretation of the data, critical revision of the subsequent versions of the manuscript, have approved the final submitted version, and agreed to be accountable for the content of the work.

## Conflict of Interest

The authors declare that the research was conducted in the absence of any commercial or financial relationships that could be construed as a potential conflict of interest.

## Publisher's Note

All claims expressed in this article are solely those of the authors and do not necessarily represent those of their affiliated organizations, or those of the publisher, the editors and the reviewers. Any product that may be evaluated in this article, or claim that may be made by its manufacturer, is not guaranteed or endorsed by the publisher.
