# Supplementary material for: Serum and Urinary Osteocalcin in Healthy 7- to 19-Year-Old Finnish Children and Adolescents
Source: Front Pediatr. 2021 Aug 24;9:610227. doi: 10.3389/fped.2021.610227 (PMC8421857; doi:10.3389/fped.2021.610227)
Supplement: Supplementary file 1 [file Data_Sheet_1.docx]

Supplementary Material

# Supplementary Figures and Tables for manuscript:

Paldánius PM et al., Serum and urinary osteocalcin in healthy 7-19- year-old Finnish children and adolescents

Supplementary Figure 1: Age and Height SD distribution per allocated robust age categories, pooled for both girls and boys. Chronological age ranges at inclusion for Girls (G) and Boys (B) per age category (indicated with mean, minimum and maximum age): 8 years (G: 7.4-9.0; B: 7.7- 8.9), 11 years (G: 10.5-12.4; B: 10.4-12.4), 14 years (G:12.5-15.4; B: 12.5-15.4), 17 years (G: 15.5- 18.4; B: 15.4- 18.1) and 19 years (G: 18.5-18.8; B: none)

Supplementary Table 1: Baseline demographics per age category for GIRLS

| Parameter by age category (range) | | | Mean | | | SD | Std. Error | 95% Confidence Interval for Mean | | Minimum | Maximum |
| --- | --- | --- | --- | --- | --- | --- | --- | --- | --- | --- | --- |
|  |  |  |  |  |  |  |  | **Lower Bound** | **Upper Bound** |  |  |
| Age (years) | **8** (7.4-9.0) | | | 8.2 | | 0.65 | 0.24 | 7.60 | 8.80 | 7.4 | 9.0 |
|  | **11** (10.5-12.4) | | | 11.6 | | 0.49 | 0.10 | 11.37 | 11.76 | 10.5 | 12.4 |
|  | **14** (12.5-15.4) | | | 13.7 | | 0.88 | 0.14 | 13.45 | 14.02 | 12.5 | 15.4 |
|  | **17** (15.5- 18.4) | | | 16.7 | | 0.80 | 0.15 | 16.43 | 17.06 | 15.5 | 18.4 |
|  | **19** (18.5-18.8) | | | 18.6 | | 0.13 | 0.06 | 18.47 | 18.81 | 18.5 | 18.8 |
| Height (cm) | | **8** (7.4-9.0) | | | 129.6 | 5.69 | 2.15 | 124.57 | 134.83 | 122.5 | 137.0 |
|  |  | **11** (10.5-12.4) | | | 148.9 | 7.42 | 1.43 | 145.94 | 151.80 | 135.5 | 165.0 |
|  |  | **14** (12.5-15.4) | | | 161.8 | 7.39 | 1.17 | 159.40 | 164.12 | 145.0 | 176.5 |
|  |  | **17** (15.5- 18.4) | | | 164.8 | 5.89 | 1.13 | 162.49 | 167.14 | 151.0 | 178.2 |
|  |  | **19** (18.5-18.8) | | | 165.2 | 4.54 | 2.03 | 159.52 | 170.80 | 160.2 | 172.0 |
| Height SD | | **8** (7.4-9.0) | | | 0.5 | 0.80 | 0.30 | -0.24 | 1.24 | -0.5 | 1.5 |
|  |  | **11** (10.5-12.4) | | | 0.4 | 1.01 | 0.19 | 0.01 | 0.81 | -1.3 | 2.8 |
|  |  | **14** (12.5-15.4) | | | 0.5 | 1.00 | 0.16 | 0.17 | 0.81 | -2.3 | 2.3 |
|  |  | **17** (15.5- 18.4) | | | 0.0 | 0.78 | 0.15 | -0.30 | 0.31 | -2.0 | 1.6 |
|  |  | **19** (18.5-18.8) | | | 0.0 | 0.81 | 0.36 | -1.03 | 0.99 | -0.9 | 1.2 |
| Weight | | **8** (7.4-9.0) | | | 28.8 | 7.18 | 2.71 | 22.20 | 35.48 | 20.9 | 39.7 |
|  |  | **11** (10.5-12.4) | | | 39.0 | 7.76 | 1.49 | 35.93 | 42.07 | 29.0 | 59.0 |
|  |  | **14** (12.5-15.4) | | | 51.0 | 11.76 | 1.86 | 47.23 | 54.75 | 27.3 | 86.8 |
|  |  | **17** (15.5- 18.4) | | | 55.7 | 8.90 | 1.71 | 52.18 | 59.22 | 36.3 | 84.6 |
|  |  | **19** (18.5-18.8) | | | 58.5 | 6.69 | 2.99 | 50.17 | 66.79 | 49.7 | 67.4 |
| BMI | | **8** (7.4-9.0) | | | 17.0 | 3.13 | 1.18 | 14.10 | 19.90 | 13.6 | 22.3 |
|  |  | **11** (10.5-12.4) | | | 17.5 | 2.50 | 0.48 | 16.51 | 18.49 | 14.2 | 24.3 |
|  |  | **14** (12.5-15.4) | | | 19.4 | 3.71 | 0.59 | 18.18 | 20.56 | 13.0 | 28.0 |
|  |  | **17** (15.5- 18.4) | | | 20.4 | 2.60 | 0.50 | 19.41 | 21.46 | 15.9 | 28.5 |
|  |  | **19** (18.5-18.8) | | | 21.4 | 1.79 | 0.80 | 19.18 | 23.62 | 19.4 | 23.4 |
| WB BMD Z score | | **8** (7.4-9.0) | | | 0.8 | 0.50 | 0.19 | 0.33 | 1.25 | 0.0 | 1.4 |
|  |  | **11** (10.5-12.4) | | | 0.2 | 0.47 | 0.09 | 0.06 | 0.43 | -1.0 | 1.3 |
|  |  | **14** (12.5-15.4) | | | 0.1 | 0.66 | 0.10 | -0.08 | 0.33 | -1.0 | 1.3 |
|  |  | **17** (15.5- 18.4) | | | 0.1 | 0.57 | 0.11 | -0.15 | 0.30 | -1.0 | 1.1 |
|  |  | **19** (18.5-18.8) | | | -0.1 | 0.54 | 0.24 | -0.75 | 0.60 | -1.0 | 0.4 |
| S-25-OH Vit D | | **8** (7.4-9.0) | | | 44.3 | 14.26 | 5.39 | 31.1 | 57.47 | 21.0 | 60.0 |
|  |  | **11** (10.5-12.4) | | | 40.1 | 11.75 | 2.26 | 35.50 | 44.79 | 19.0 | 61.0 |
|  |  | **14** (12.5-15.4) | | | 37.7 | 9.86 | 1.56 | 34.55 | 40.85 | 18.0 | 63.0 |
|  |  | **17** (15.5- 18.4) | | | 46.0 | 16.43 | 3.22 | 39.32 | 52.60 | 18.0 | 82.0 |
|  |  | **19** (18.5-18.8) | | | 42.8 | 12.97 | 5.80 | 26.70 | 58.90 | 29.0 | 61.0 |
| PTH | | **8** (7.4-9.0) | | | 45.2 | 25.07 | 10.24 | 18.86 | 71.48 | 19.0 | 79.0 |
|  |  | **11** (10.5-12.4) | | | 43.7 | 13.90 | 2.67 | 38.17 | 49.16 | 20.0 | 78.0 |
|  |  | **14** (12.5-15.4) | | | 54.4 | 27.08 | 4.45 | 45.38 | 63.44 | 17.0 | 135.0 |
|  |  | **17** (15.5- 18.4) | | | 37.5 | 15.37 | 3.01 | 31.33 | 43.74 | 14.0 | 87.0 |
|  |  | **19** (18.5-18.8) | | | 41.8 | 11.08 | 4.95 | 28.05 | 55.55 | 27.0 | 54.0 |
| WB total fat% | | **8** (7.4-9.0) | | | 29.8 | 9.13 | 3.45 | 21.40 | 38.28 | 20.5 | 44.4 |
|  |  | **11** (10.5-12.4) | | | 29.0 | 6.48 | 1.25 | 26.44 | 31.57 | 13.6 | 40.5 |
|  |  | **14** (12.5-15.4) | | | 28.4 | 8.15 | 1.29 | 25.78 | 31.00 | 17.1 | 49.8 |
|  |  | **17** (15.5- 18.4) | | | 29.8 | 5.33 | 1.26 | 27.12 | 32.42 | 19.4 | 37.7 |
|  |  | **19*** (18.5-18.8) | | | - | - | - | - | - | - | - |

**Whole Body (WB) fat% missing from all GIRLS in age category 19*

Supplementary Table 2: Baseline demographics per age category for BOYS

| Parameter by age category (range) | | Mean | | SD | Std. Error | 95% Confidence Interval for Mean | | | Minimum | Maximum |
| --- | --- | --- | --- | --- | --- | --- | --- | --- | --- | --- |
|  |  |  |  |  |  | **Lower Bound** | | **Upper Bound** |  |  |
| Age | **8** (7.7- 8.9) | | 8.6 | 0.45 | 0.13 | 8.28 | 8.85 | | 7.7 | 8.9 |
|  | **11** (10.4-12.4) | | 11.4 | 0.60 | 0.13 | 11.10 | 11.63 | | 10.4 | 12.4 |
|  | **14** (12.5-15.4) | | 13.6 | 0.94 | 0.22 | 13.11 | 14.05 | | 12.5 | 15.4 |
|  | **17** (15.4- 18.1) | | 16.5 | 0.74 | 0.20 | 16.09 | 16.98 | | 15.6 | 18.1 |
| Height | **8** (7.7- 8.9) | | 131.7 | 6.29 | 1.81 | 127.71 | 135.70 | | 118.5 | 141.5 |
|  | **11** (10.4-12.4) | | 147.5 | 6.90 | 1.44 | 144.52 | 150.48 | | 129.5 | 161.5 |
|  | **14** (12.5-15.4) | | 162.9 | 11.70 | 2.76 | 157.08 | 168.72 | | 147.0 | 183.5 |
|  | **17** (15.4- 18.1) | | 178.2 | 10.67 | 2.96 | 171.76 | 184.65 | | 160.0 | 199.9 |
| Height SD | **8** (7.7- 8.9) | | 0.4 | 1.13 | 0.33 | -0.34 | 1.09 | | -1.3 | 2.3 |
|  | **11** (10.4-12.4) | | 0.5 | 1.02 | 0.21 | 0.08 | 0.96 | | -1.8 | 2.9 |
|  | **14** (12.5-15.4) | | 0.6 | 0.85 | 0.20 | 0.20 | 1.04 | | -0.8 | 2.1 |
|  | **17** (15.4- 18.1) | | 0.4 | 1.43 | 0.40 | -0.45 | 1.27 | | -1.6 | 3.2 |
| Weight | **8** (7.7- 8.9) | | 30.1 | 4.57 | 1.32 | 27.21 | 33.02 | | 20.7 | 37.1 |
|  | **11** (10.4-12.4) | | 40.2 | 7.30 | 1.52 | 37.01 | 43.32 | | 26.4 | 54.3 |
|  | **14** (12.5-15.4) | | 55.1 | 16.72 | 3.94 | 46.79 | 63.42 | | 36.3 | 96.5 |
|  | **17** (15.4- 18.1) | | 70.3 | 15.31 | 4.25 | 61.06 | 79.57 | | 53.5 | 113.0 |
| BMI | **8** (7.7- 8.9) | | 17.3 | 1.98 | 0.57 | 16.04 | 18.56 | | 14.7 | 21.8 |
|  | **11** (10.4-12.4) | | 18.3 | 2.32 | 0.48 | 17.33 | 19.34 | | 14.0 | 22.3 |
|  | **14** (12.5-15.4) | | 20.4 | 5.01 | 1.21 | 17.87 | 23.02 | | 15.6 | 34.8 |
|  | **17** (15.4- 18.1) | | 22.1 | 3.88 | 1.07 | 19.74 | 24.42 | | 17.7 | 32.7 |
| WB BMD Z score | **8** (7.7- 8.9) | | 0.7 | 0.60 | 0.17 | 0.29 | 1.06 | | 0.0 | 1.6 |
|  | **11** (10.4-12.4) | | 0.3 | 0.66 | 0.14 | 0.05 | 0.62 | | -1.0 | 1.8 |
|  | **14** (12.5-15.4) | | 0.1 | 0.55 | 0.13 | -0.16 | 0.37 | | -1.0 | 1.4 |
|  | **17** (15.4- 18.1) | | 0.1 | 0.48 | 0.13 | -0.14 | 0.43 | | -1.0 | 0.9 |
| S-25-OH Vit D | **8** (7.7- 8.9) | | 48.2 | 7.93 | 2.29 | 43.13 | 53.20 | | 39.0 | 69.0 |
|  | **11** (10.4-12.4) | | 45.8 | 10.35 | 2.16 | 41.35 | 50.30 | | 26.0 | 65.0 |
|  | **14** (12.5-15.4) | | 43.1 | 14.05 | 3.31 | 36.12 | 50.1 | | 17.0 | 77.0 |
|  | **17** (15.4- 18.1) | | 42.8 | 12.56 | 3.48 | 35.18 | 50.36 | | 26.0 | 69.0 |
| PTH | **8** (7.7- 8.9) | | 37.4 | 16.41 | 4.74 | 26.99 | 47.84 | | 20.0 | 83.0 |
|  | **11** (10.4-12.4) | | 41.7 | 18.31 | 3.82 | 33.82 | 49.66 | | 8.0 | 78.0 |
|  | **14** (12.5-15.4) | | 51.1 | 33.23 | 8.06 | 34.03 | 68.20 | | 14.0 | 136.0 |
|  | **17** (15.4- 18.1) | | 35.5 | 12.75 | 3.53 | 27.76 | 43.16 | | 14.0 | 51.0 |
| Whole body total fat% | **8** (7.7- 8.9) | | 25.2 | 5.75 | 1.66 | 21.57 | 28.88 | | 17.8 | 37.0 |
|  | **11** (10.4-12.4) | | 26.1 | 6.20 | 1.29 | 23.45 | 28.81 | | 15.2 | 39.9 |
|  | **14** (12.5-15.4) | | 24.2 | 9.46 | 2.23 | 19.54 | 28.95 | | 9.5 | 43.5 |
|  | **17** (15.4- 18.1) | | 18.7 | 10.36 | 3.12 | 11.71 | 25.63 | | 9.7 | 43.7 |

Supplementary Table 3: Dependent variables and regression model coefficients

|  | **Total serum OC** | | | **cOC** | | | **Urinary mid-OC** | | |
| --- | --- | --- | --- | --- | --- | --- | --- | --- | --- |
|  | ***Beta_std_*** | ***95% CI*** | ***p value*** | ***Beta_std_*** | ***95% CI*** | ***p value*** | ***Beta_std_*** | ***±-95% CI*** | ***p value*** |
| constant | - | -162.5, 36.1 | 0.210 | **-** | -183.9, 23.6 | 0.129 | - | -49.2, 63.2 | 0.807 |
| ***Age*** | **-1.007** | -7.464, -2.739 | **<0.001** | -**1.083** | -8.448, -3.509 | **<0.001** | **-0.631** | -3.280, -0.610 | **0.005** |
| ***Height*** | **1,354** | 0.407, 1.953 | **0.003** | **1.454** | 0.573, 2.188 | **0.001** | 0.516 | -0.160, 0.703 | 0.216 |
| ***Weight*** | -1.043 | -1.841, 0.033 | 0.059 | **-1.105** | -2.023, -0.065 | **0.037** | -0.197 | -0.633, 0.425 | 0.697 |
| Sex | -0.035 | -5.676, 3.943 | 0.722 | -0.032 | -5.902, 4.149 | 0.731 | 0.136 | -0.606, 4.793 | 0.127 |
| Height SD | -0.175 | -5.973, 1.640 | 0.262 | -0.236 | -7.166, 0.789 | 0.115 | -0.161 | -3.403, 0.898 | 0.251 |
| Weight % | 0.116 | -0.337, 0.502 | 0.699 | 0.033 | -0.413, 0.464 | 0.908 | 0.042 | -0.215, 0.252 | 0.874 |
| BMI | 0.449 | -1.810, 5.042 | 0.353 | 0.593 | -1.257, 5.903 | 0.202 | -0.168 | -2.326, 1.575 | 0.704 |
| ***Puberty*** | 0.002 | -3.575, 3.641 | 0.986 | -0.097 | -5.273, 2.267 | 0.432 | **-0.272** | -4.525, -0.233 | **0.030** |
| ***PTH*** | 0.144 | -0.002, 0.159 | 0.054 | **0.163** | 0.013, 0.181 | **0.023** | 0.085 | -0.017, 0.075 | 0.216 |
| 25-OH Vit D | -0.135 | -0.280, 0.014 | 0.076 | -0.099 | -0.260, 0.047 | 0.174 | 0.006 | -0.079, 0.087 | 0.927 |
| WB fat% | -0.208 | -0.724, 0.081 | 0.116 | -0.209 | -0.772, 0.069 | 0.101 | 0.098 | -0.131, 0.319 | 0.409 |
| WB Z score | -0.103 | -6.975, 2.824 | 0.404 | -0.010 | -5.340, 4.899 | 0.932 | 0.112 | -1.392, 4.231 | 0.319 |
| Femur Z score | -0.016 | -3.574, 3.091 | 0.886 | -0.106 | -5.189, 1.777 | 0.334 | -0.131 | -2.089, 1.829 | 0.896 |
| LS Z score | 0.003 | -3.429, 3.515 | 0.981 | 0.029 | -3.195, 4.062 | 0.814 | -0.056 | -2.496, 1.513 | 0.628 |
